# A patient with positive anti‐IFN‐γ autoantibody and monoclonal gammaglobulinemia masquerading as multiple myeloma: Case report and literature review

**DOI:** 10.1002/ccr3.9446

**Published:** 2024-09-18

**Authors:** Ran An, Zhiyin Liu, Fangxiu Luo, Zeying Yan, Ying Wang, Haimin Sun, Jie Tian, Yu Chen, Yubao Chen

**Affiliations:** ^1^ Department of Hematology, Ruijin Hospital Shanghai Jiao Tong University School of Medicine Shanghai China; ^2^ Department of Pathology, Ruijin Hospital Shanghai Jiao Tong University School of Medicine Shanghai China

**Keywords:** adult‐onset immunodeficiency, anti‐IFN‐γ autoantibody, monoclonal gammaglobulinemia, multiple myeloma, non‐tuberculous mycobacterial infection

## Abstract

**Key Clinical Message:**

Adult‐onset immunodeficiency (AOID) is an emerging acquired immunodeficiency, characterized by multiple opportunistic infections including non‐tuberculous mycobacterium (NTM) due to the presence of anti‐IFN‐γ autoantibody (AIGA). This case highlights the challenges of accurate diagnosis of monoclonal gammaglobulinemia with NTM infection and favorable outcomes of anti‐plasma cell therapy in AOID.

**Abstract:**

Adult‐onset immunodeficiency (AOID) is an emerging acquired immunodeficiency due to anti‐IFN‐γ autoantibody (AIGA) with low morbidity, frequent disseminated infections, a prolonged course, difficult diagnosis and treatment, and a poor prognosis. Here, we report a patient with positive AIGA and monoclonal gammaglobulinemia who was mimicking symptomatic multiple myeloma and resulting in a non‐tuberculous mycobacterial (NTM) infection. While he achieved an excellent therapeutic effect with anti‐plasma cell therapy, it also serves as a warning that monoclonal gammaglobulinemia with NTM infection is easily misdiagnosed as symptomatic multiple myeloma, and the screening for AIGA should not be ignored in patients with NTM infection.

## 1 INTRODUCTION

Anti‐interferon‐γ antibody‐associated adult‐onset immunodeficiency (AIGA‐AOID) is a rare adult‐onset immunodeficiency disease,^1^, ^2^ which is challenging to diagnose and prone to underdiagnosis and misdiagnosis due to its low incidence, atypical clinical manifestations, and unavailability of screening methods. Its clinical manifestation was various and featured by severe disseminated infections with intracellular pathogens, especially non‐tuberculous mycobacterial (NTM). *Mycobacterium avium* complex (MAC) is the most prevalent non‐tuberculous mycobacterium, typically associated with pulmonary disease. However, it can also manifest as disseminated disease, presenting with a range of symptoms including fever, lymphadenopathy, skin lesions, bone destruction, diarrhea, and so forth.^3^ Currently, the diagnosis and treatment of AIGA‐AOID are only based on case reports and small‐scale exploratory studies, and there are very few literature reports on AIGA‐AOID associated with malignant hematologic diseases.

In this paper, we report a case of a patient with MGUS combined with AOID leading to NTM infection, with clinical manifestations of early leukocytosis, enlarged lymph nodes, anemia, bone destruction, and M protein positivity, which was misdiagnosed as symptomatic multiple myeloma (MM). To our knowledge, this is the first case report of malignant plasma cell disease with AIGA‐AOID. By presenting the diagnosis and management of this patient and reviewing the literature, we aim to improve clinicians' knowledge of the differential diagnosis of such patients and avoid misdiagnosis and underdiagnosis.

## 2 CASE HISTORY AND EXAMINATION

A 52‐year‐old male patient was admitted to our clinic on February 12, 2023, with enlarged right cervical lymph nodes and bone pain for about 1 month. He had been diagnosed with silicosis in 2019, although he reported that he had no environmental or occupational exposures.

**FIGURE 1:**
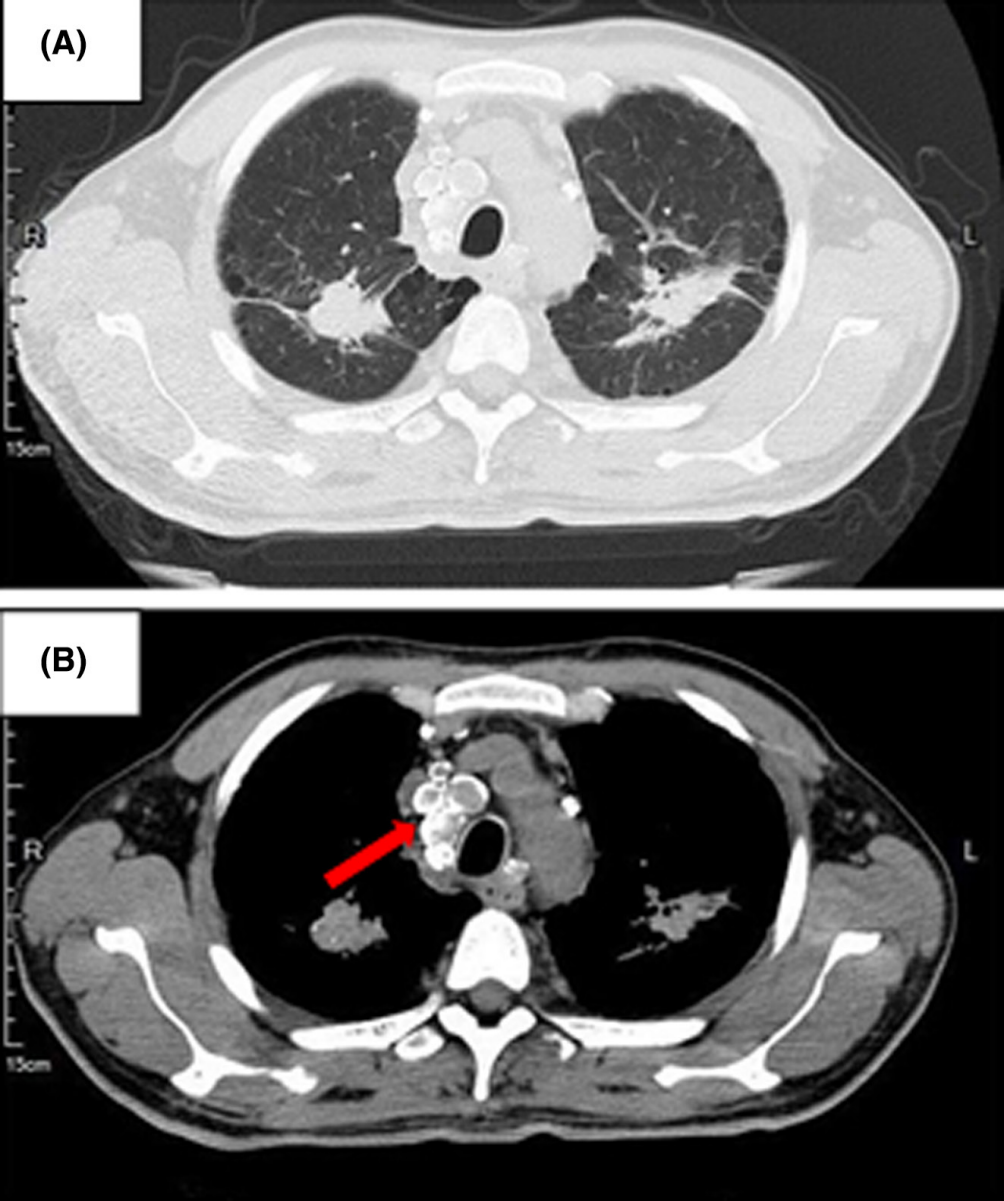
Chest CT imaging. (A) Multiple high‐density shadows of upper pulmonary lobe. (B) Red arrows indicate sites of multiple enlarged lymph nodes with calcification.

**FIGURE 2:**
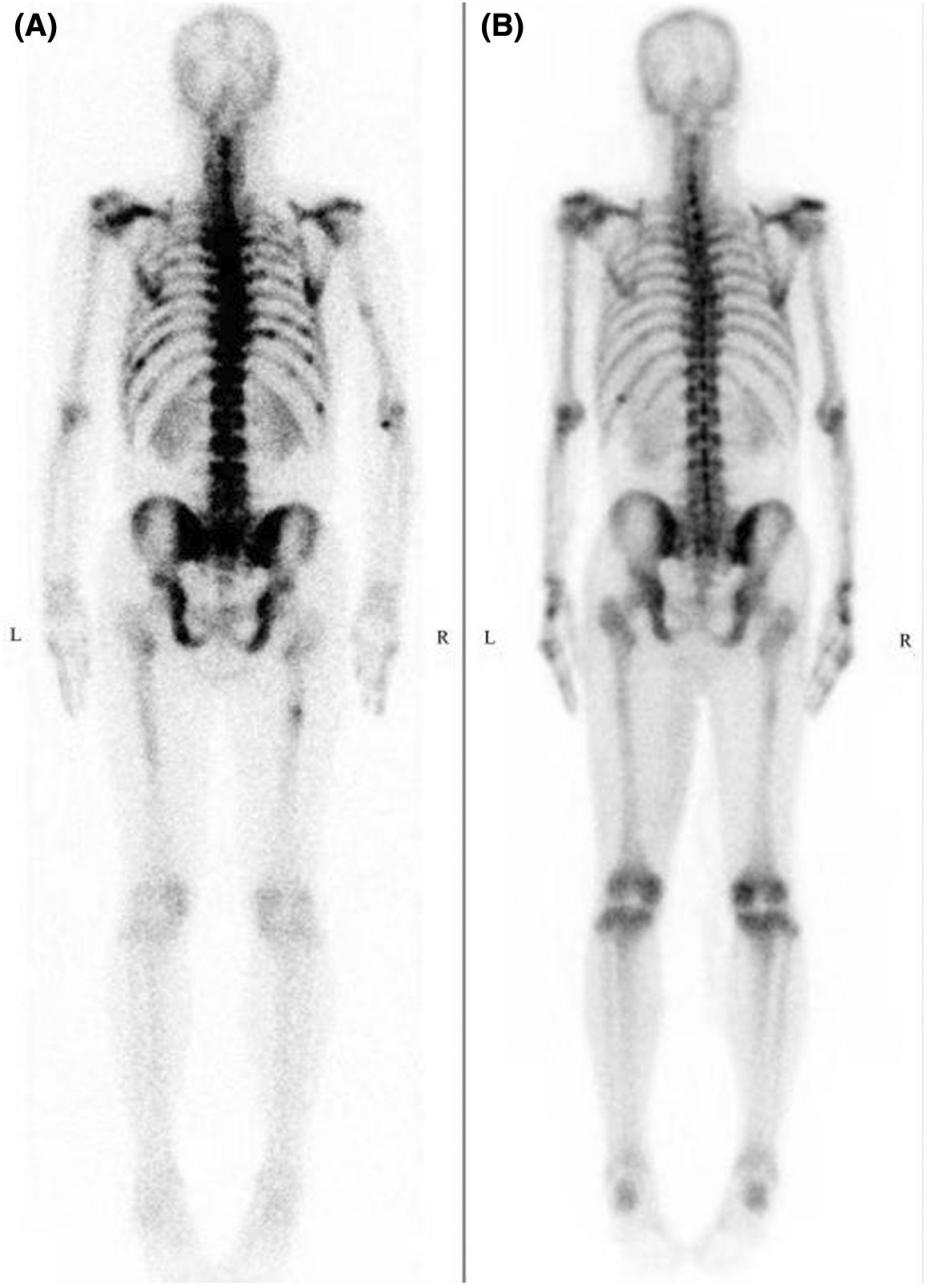
Isotope bone scan. (A) Multiple radioactive concentrations in the skeleton in February 2023. (B) Significant reduction in skeletal lesions compared to February 2023.

Blood levels of coagulation function, ferritin, folic acid, and vitamin B12 were normal. Serum tests for Coomb's test were negative, as was the PNH clone. Antinuclear antibody profile showed positive for p‐ANCA, and other laboratory test results are shown in Table 1. Computed tomography (CT) of the chest, performed after the administration of intravenous contrast material, showed interstitial fibrosis and multiple high‐density shadows in both lung and multiple enlarged lymph nodes with calcification in the mediastinum (Figure 1A,B). A systemic bone X‐ray revealed a slight lateral curvature of the thoracic vertebrae and multiple peripheral calcified nodules in the mediastinum and hilum. An isotope bone scan revealed multiple radioactive concentrations in the left eyebrow arch, bilateral scapulae, bilateral femurs, sternum, spine, pelvis, and right humerus (Figure 2A). Ultrasonography showed multiple enlarged lymph nodes in the bilateral neck, axilla, and groins. Pathology of puncture biopsy of the right cervical lymph node was suggestive of granulomatous lesions of lymphoid tissue, with histiocytes and more neutrophilic hyperplasia. A bone marrow biopsy conducted in February 2023 showed a striking rise in the neutrophil alkaline phosphatase (NAP) score, and plasma cells accounted for 2.5%. Meanwhile, flow cytometry demonstrated that positive for CD138, CD38, CD56, CD117, CD81, CD27, CD45, and negative for CD19, and kappa light chain restricted expression. Subsequently, cytogenetic analysis further revealed the deletion of RB1 and D13S319. The molecular profiling of the gene panel was normal.

**TABLE 1:** Laboratory results of the patient.

	Reference range	February 2023	April 2023	June 2023
WBC (*10^9^/L)	3.5–9.54	39.84	21.38	6.48
Neutrophil (*10^9^/L)	1.8–6.3	32.09	16.39	5.07
HB (g/L)	130–175	95	73	51
PLT (*10^9^/L)	125–350	370	220	9
CRP (mg/L)	<3	117	62.2	80.9
TP (g/L)	66–83	85	79	50
ALB (g/L)	35–52	27	20	22
Creatinine (μmol/L)	72–127	91	87	114
Ca (mmol/L)	2.2–2.65	2.07	2.67	2.41
LDH (U/L)	<248	273	102	148
β2MG (ng/mL)	1090–2530	5060	10,400	1230
Immunofixation electrophoresis (IFE)		IgA‐k	IgA‐k	IgA‐k
Monoclonal protein concentrations (g/L)		27.09	22.1	17.9
IgG (g/L)	8.6–17.4	21	19.2	14.8
IgA (g/L)	1.0–4.2	16.4	5.11	4.3
IgM (g/L)	0.3–2.2	0.26	0.31	0.32
sFLC‐κ (mg/L)	6.7–22.4	128	163	246
sFLC‐λ (mg/L)	8.3–27	120	124	161
24‐h urine protein (mg)	24–150	694	378	102

## 3 DIFFERENTIAL DIAGNOSIS, INVESTIGATIONS, AND TREATMENT

This patient was diagnosed with multiple myeloma with leukemia‐like reaction, lymphadenitis, and silicosis. After treatment with the RCD regimen (lenalidomide 25 mg p.o. days 1–21, cyclophosphamide 800 mg i.v. day 1, dexamethasone 20 mg i.v. days 1–4, 8–9) chemotherapy and anti‐infectious therapy, he achieved relief of bone pain and lymph node enlargement and improvement of blood picture.

**FIGURE 3:**
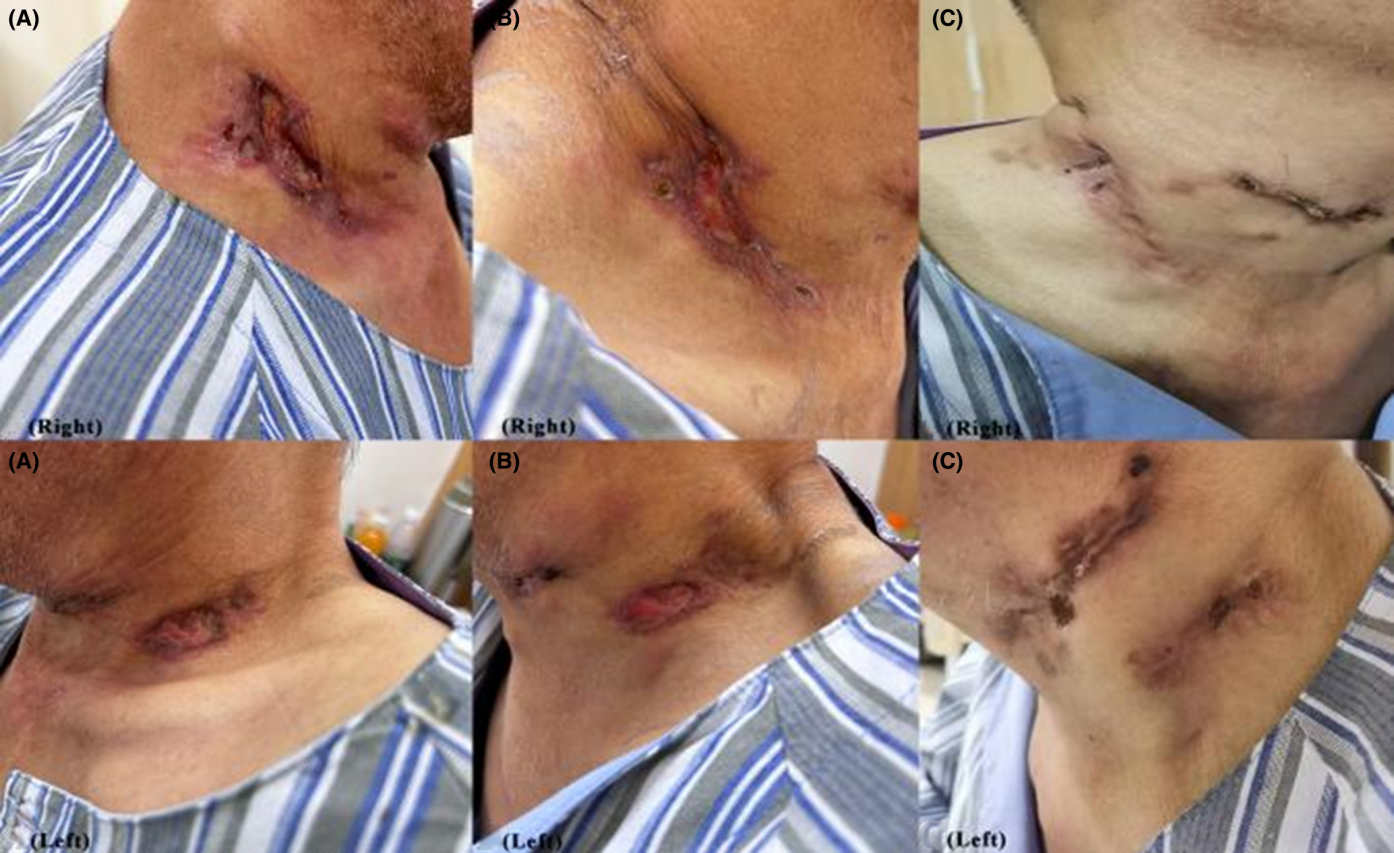
Comparative of neck lymph nodes suppurative rupture. (A) Multiple rupture and enlarge lymph nodes in the bilateral neck with pus in April 2023. (B) After 2 months of anti‐NTM treatment in June 2023. (C) Ruptured lymph nodes have healed and old scars are visible in December 2023.

**FIGURE 4:**
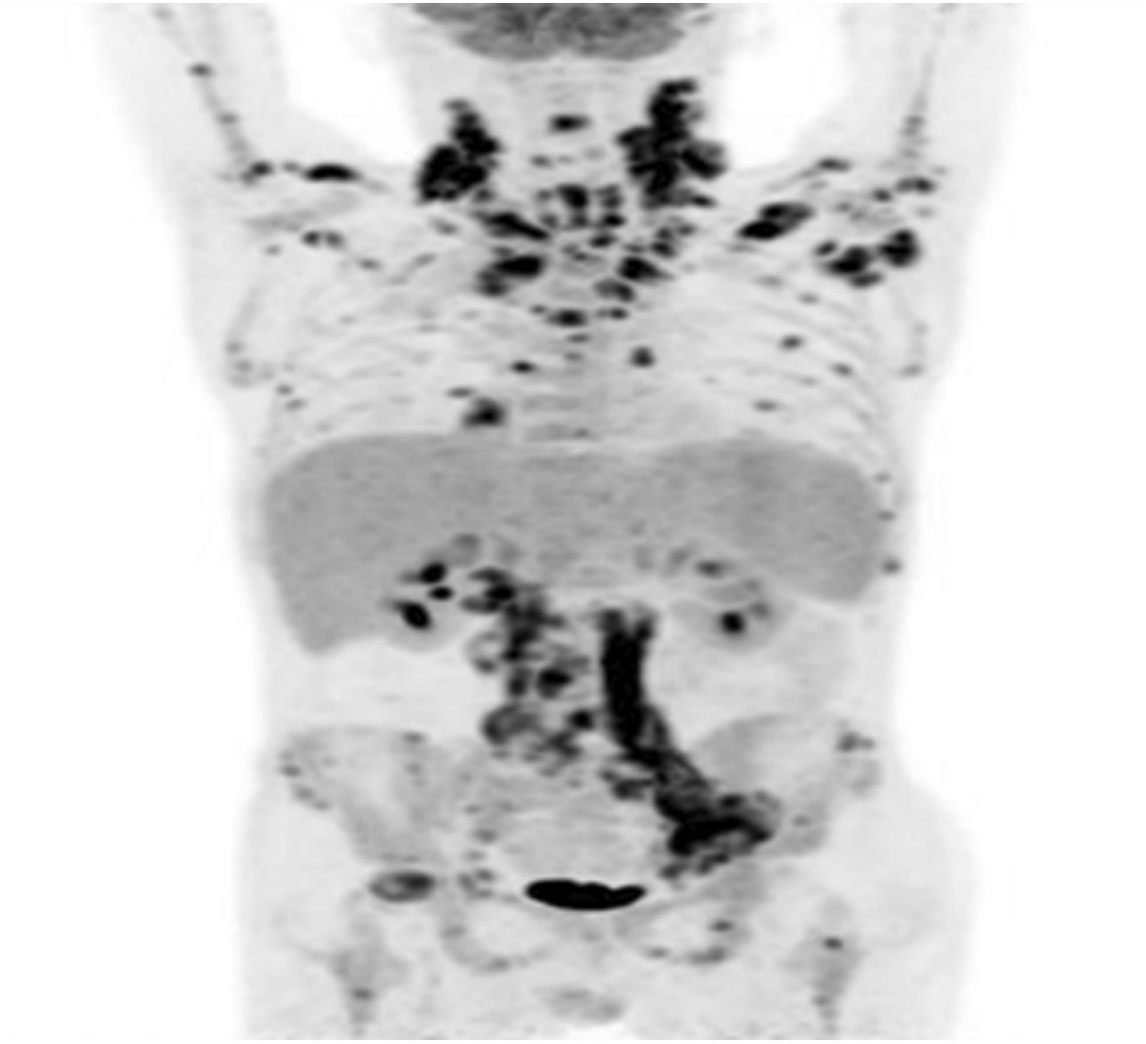
PET/CT image shows increased FDG uptake in multiple parts of the skeleton, multiple lymph nodes, and multiple irregular mass of upper pulmonary lobe.

**FIGURE 5:**
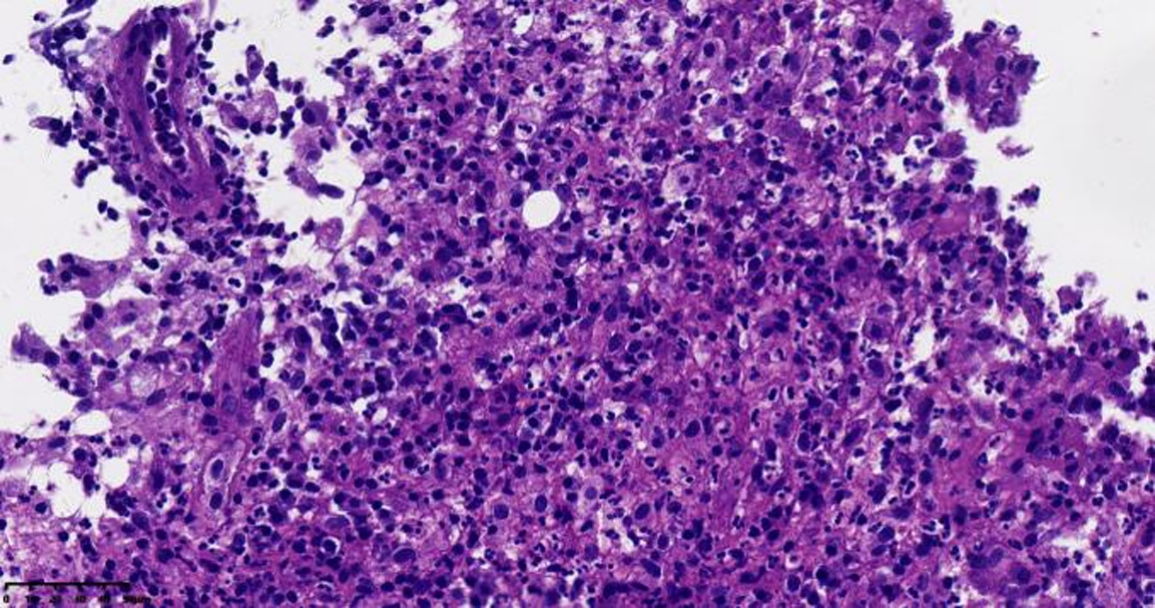
Biopsy pathology of inguinal lymph node: Chronic inflammation with granulation tissue hyperplasia. Microscopic observation: new capillaries, fibroblasts, and infiltrated with not a few inflammatory cells (HE X100).

**FIGURE 6:**
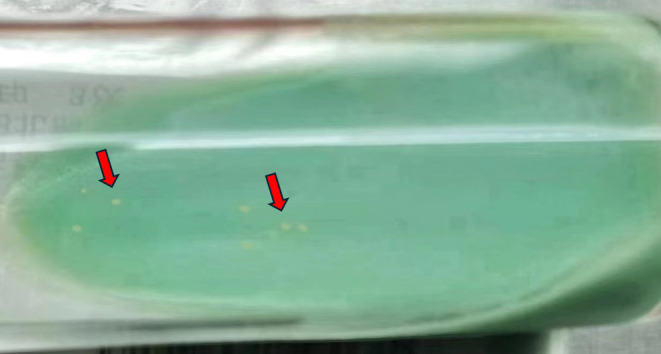
Cultivate mycobacterium tuberculosis in the pus of ruptured lymph nodes. Following a period of 41 days, the growth of small colonies of *M. tuberculosis* can be observed on solid Roche medium (red arrows).

In April 2023, the patient was readmitted to the hospital with a fever and enlarged ruptured suppurative lymph nodes. On examination, the temporal temperature was 38.1°C, the heart rate 102 beats per min, the blood pressure 120/90 mmHg. The patient appeared anemic appearance, and multiple ruptured and enlarged lymph nodes were seen in the right groin and bilateral neck with pus (Figure 3A). Laboratory tests showed calcitoninogen was 1.02 ng/mL (reference value 0–0.5 ng/mL). Serum tests for cryptococcal antigen, 1,3‐β‐d‐glucan, and HIV were negative, and other laboratory test results are shown in Table 1. Magnetic resonance imaging of the lumbar spine revealed degenerative alterations in the thoracolumbar spine, characterized by numerous abnormal signal centers in certain vertebral bodies, and localized swelling in the specified attachments and adjacent soft tissues. Fluorine18‐FDG PET/CT images showed high uptake of the radiopharmaceutical in multiple parts of the skeleton (SUV max = 7.1), multiple lymph nodes (SUV max = 9.2), and multiple irregular mass of upper pulmonary lobe (SUV max = 1.7) (Figure 4). Conducting another bone marrow aspiration revealed that the NAP activity of his posterior iliac bone marrow smear remained elevated, and the bone marrow cultures exhibited no growth. Repeated puncture biopsies of the left inguinal lymph node and pathology indicated granulation and fibrous tissue with acute and chronic inflammation of blood vessels (Figure 5), negative for EBV‐encoded RNA in situ hybridization (EBER‐ISH), acid‐fast staining, and tuberculosis DNA by real‐time fluorescent polymerase chain reaction (RT‐PCR). Separate sputum and pus next‐generation sequencing (NGS) examination were performed to clarify the presence of specific pathogenic infection further. The sputum NGS results suggested *Stenotrophomonas maltophilia* with a sequence number of 380, *Klebsiella pneumoniae* with a sequence number of 23, *Candida tropicalis* with a sequence number of 107903, human herpesvirus with a sequence number of 1358, and *M. avium* with a sequence number of 75. The pus NGS results suggested intracellular *M. avium* with a sequence number of 684. Positive results were observed in the pus smear, as was the *M. tuberculosis* culture after 41 days (Figure 6), whereas the Xpert MTB/RIF (Xpert) test of pus was negative. Thus, the patient was clearly diagnosed with NTM infection. Therefore, we gave a combination of clarithromycin, moxifloxacin, ethambutol, and rifapentine for NTM infection treatment and added albumin and intravenous immunoglobulin (IVIG) due to immune deficiency. Furthermore, we did dressing change with amikacin sulfate injection and silver sulfadiazine cream to manage the ulcerated lymph nodes.

On June 14, 2023, the patient revisited the hospital because of persistent tiredness and dizziness lasting for 1 week. Laboratory evaluation revealed liver function and kidney function were normal, as were the blood levels of electrolytes and glucose. However, the blood level of hemoglobin had decreased to 51 g/L, and the platelet count dropped to 17 × 10^9^/L. We tested the patient's plasma for AIGA and found AIGA titer of 1:2500. An isotope bone scan showed a notable decrease in bone lesions compared to February 2023 (Figure 2B). So far, a correct diagnosis in this case is monoclonal gammaglobulinemia of undetermined significance (MGUS) with AIGA‐AOID, and bortezomib, a proteosome inhibitor, was added to his treatment regimen in order to target reduces the generation of AIGA. The regimen consists of bortezomib 1.3 mg/m^2^ subcutaneously and dexamethasone 20 mg intravenous on days 1, 4, 8, and 11.

## 4 OUTCOME AND FOLLOW‐UP

As of submission, the patient continues to receive standard anti‐NTM treatment, clarithromycin 0.5 g/day, moxifloxacin 0.4 g/day, ethambutol 0.75 g/day, rifampicin 0.45 g twice a week, and the ruptured neck lymph nodes have recovered (Figure 3A–C) and survived usually.

## 5 DISCUSSION

AOID with acquired AIGA was first reported by German scientists in 2004,^4^ with an incidence of about 0.5–1.0 per million people. AIGA can inhibit IFN‐γ‐STAT‐1 phosphorylation and downregulate the production of its downstream factors (IL‐12 and TNF‐α), causing severe defects in the immune response of Th1 cells, resulting in impaired clearance of intracellular pathogens and susceptibility to severe or disseminated opportunistic infections, with an inferior prognosis.^5^ Several studies have found that AIGA‐positive patients are mostly Southeast Asians, and European and American patients are mostly Asian immigrants, suggesting that there are geographical differences in the disease. HLA phenotypes such as HLA‐DRB1 and HLA‐DQB1 are considered to be associated with the disease.^6^ Our patient had a history of silicosis, and studies have shown that the development of silicosis is closely related to the suppression of cellular immune function and complex cytokine interactions,^7^, ^8^ so it cannot be excluded that the production of AIGA in this patient was related to a history of silicosis. As for the development of MGUS in this patient, we hypothesized that the long‐term chronic irritation caused by the patient's silicosis and infection led to the emergence of malignant plasma cell clone.

Literature reports show that common infectious pathogens in patients with AIGA‐AOID clinically include NTM, Malniferous Basketball Bacteria, Varicella zoster virus, and *Salmonella* spp. In patients with previous healthy adult immunodeficiency, AIGA is closely associated with NTM infection, in which bird‐intracellular mycobacterium complex (*M. avium* complex, MAC) is one of the common strains in NTM, accounting for 42.8%–81.6%. Disseminated NTM infections can involve multiple organs, including lungs, lymph nodes, skin soft tissues, bones, etc., and their clinical manifestations are diverse.^1^, ^3^, ^9^ Our patient started with hyperleukocytosis and positive immunofixation electrophoresis with anemia, bone destruction, and multiple lymph node enlargement. The clinical diagnosis of this case in the first stage was multiple myeloma with leukemia‐like reaction, lymphadenitis, and silicosis. The patient's symptoms of bone pain and anemia were improved after one course of treatment with the RCD regimen, but fever and suppurative changes of lymph nodes appeared soon afterward. Sputum and pus NGS tests were sent for examination, and it was clear that the infection was caused by MAC. The constellation of symptoms of disseminated MAC infection, lymphadenopathy, and HIV‐negative raised suspicion for an immunodeficiency syndrome. Further serum anti‐INF‐γ autoantibody test finally confirmed the antibody existence that led to his immunodeficiency. Studies have shown that MAC is the most common causative NTM subspecies of bone disseminated NTM infection, mainly as osteolytic lesions, pathological fractures, etc. The possible mechanism is that the balance between osteoclasts and osteoblasts is disrupted due to the blocking and neutralizing effects of AIGA against IFN‐γ,^10^, ^11^ and it is elementary to misdiagnose it as bone tuberculosis, metastatic tumors, and multiple myeloma. It is generally recognized that skeletal lesions of multiple myeloma are more difficult to improve in the short term. Although no biopsy of the bony lesion was performed in this case, the bone destruction of multiple positions was significantly reduced after anti‐NTM treatment. So, we inferred that the skeletal involvement was caused by *M. avium* infection, and MM diagnosis was misled.

There is no consensus on the treatment of anti‐IFN‐γ antibody immunodeficiency syndromes, and evidence‐based medical evidence suggests that treatment to lower AIGA titers is needed in addition to anti‐infective therapy.^3^, ^12^ Previous studies have shown that rituximab eliminates antibody‐producing B cells, decreases AIGA titers, and restores immune function mediated by endogenous IFN‐γ.^13^ The benefit of rituximab has also been demonstrated in treating some patients with anti‐IFN‐γ antibody immunodeficiency syndrome.^14^, ^15^ In addition, immunosuppressive drugs such as cyclophosphamide, which reduces AIGA production through its inhibitory effect on B cells and T cells, have also been effective in a few cases.^16^, ^17^ Recently, anti‐plasma cell therapy, bortezomib or anti‐CD38 monoclonal antibody, has been reported to be an effective treatment for NTM infections, significantly improving the clinical status of patients.^18^, ^19^, ^20^ Other therapeutic strategies, including subcutaneous injection of IFN‐γ and intravenous IVIG, have also been explored, but their effectiveness remains verified.^21^ Due to the diagnosis of multiple myeloma in the first stage, our patient's lymph node enlargement and bone pain symptoms improved after treatment with a regimen containing lenalidomide and cyclophosphamide. It was later clarified as disseminated *M. avium* infection, and there was still a progressive decrease in blood counts during regular anti‐NTM therapy; myelosuppression caused by higher titer of AIGA could not be excluded. Considering the patient with MGUS, we chose a bortezomib‐based treatment regimen and anti‐NTM therapy, which took into account both anti‐plasmacytosis medicine and lowering of AIGA titer. After treatment, the patient's blood picture improved, and the multiple bone destructions and enlarged lymph nodes were significantly reduced and alleviated. As of submission, the patient continues to be treated with clarithromycin, moxifloxacin, ethambutol, and rifapentine in anti‐NTM therapy.

Although our case achieved an excellent therapeutic effect, it still gives us a warning: In clinical practice, if we encounter malignant plasma cell disease with bone destruction, anemia, fever, recurrent lymph node enlargement, we should avoid being disturbed by other complications of MM. Once the effect of routine anti‐infection is not good, we should be alert to atypical pathogen infection, and patients with disseminated NTM infection as the manifestation should being pay attention to the screening of AIGA. It should be highlighted that NGS has a high sensitivity and fast diagnostic rate in NTM diagnosis, which can provide an essential direction for clinical diagnosis.

## 6 CONCLUSION

To our knowledge, we reported the first case of malignant plasma cell disease with anti‐IFN‐γ antibody immunodeficiency syndromes that was successfully treated with anti‐plasma cell therapy, showing that such strategy was safe and effective in AOID. Our conviction is that increasing awareness is essential to strengthen clinical suspicion, diagnosis, and effective management.

## AUTHOR CONTRIBUTIONS

**Ran An:** Conceptualization; data curation; writing – original draft. **Zhiyin Liu:** Conceptualization; data curation; supervision; writing – review and editing. **Ying Wang:** Conceptualization; data curation; writing – review and editing. **Fangxiu Luo:** Data curation; methodology; validation; visualization. **Zeying Yan:** Conceptualization; data curation; writing – review and editing. **Haimin Sun:** Conceptualization; data curation; writing – review and editing. **Jie Tian:** Conceptualization; data curation; writing – review and editing. **Yu Chen:** Supervision; validation; writing – original draft; writing – review and editing. **Yubao Chen:** Conceptualization; project administration; supervision; validation; writing – original draft; writing – review and editing.

## FUNDING INFORMATION

This research did not receive any specific grant from funding agencies in the public, commercial, or not‐for‐profit sectors.

## CONFLICT OF INTEREST STATEMENT

The authors declare no conflicts of interest.

## ETHICS STATEMENT

Written informed consent was obtained from the patient for publication of this case report and of the associated images.

## CONSENT

Written informed consent was obtained from the patient to publish this report in accordance with the journal's patient consent policy.

## Data Availability

The authors confirm that the data supporting the findings of this study are available within the article.
